# Conventional Ameloblastoma. A Case Report with Microarray and Bioinformatic Analysis

**DOI:** 10.3390/diagnostics12123190

**Published:** 2022-12-16

**Authors:** Emiliano Jurado-Castañeda, Carla Monserrat Ramírez-Martínez, Alejandro Alonso-Moctezuma, Jessica Tamara Páramo-Sánchez, Diana Ivette Rivera-Reza, Osmar Alejandro Chanes-Cuevas, César Luis Ortiz-Solís, Mario Alberto Téliz-Meneses, Oscar Rohel Hernández-Ortega, Marco Xavier Vizzuete-Bolaños, Patricio Olmedo-Bastidas, Luis Fernando Jacinto-Alemán

**Affiliations:** 1 Department of Oral Medicine and Pathology, Postgraduate Division, Dental School, National Autonomous University of Mexico, Mexico City 04510, Mexico; 2 Oral and Maxillofacial Surgery Specialty, Postgraduate Division, Dental School, National Autonomous University of Mexico, Mexico City 04510, Mexico; 3 Dental Biomaterials Laboratory, Postgraduate Division, Dental School, National Autonomous University of Mexico, Mexico City 04510, Mexico; 4 Maxillofacial Surgery Department, General Hospital of Balbuena, Mexico City 15970, Mexico

**Keywords:** ameloblastoma, odontogenic tumor, hemimandibulectomy, microarray analysis, bioinformatics

## Abstract

Ameloblastoma is a rare benign epithelial odontogenic neoplasm, but with great clinical implications, as despite its benignity and slow growth, most cases are locally aggressive with a significant recurrence rate. Histological, cellular, or molecular analyses of its pathogenesis have confirmed the complexity of this neoplasm. We present the case of a 20-year-old patient with a suggestive clinical and radiographic diagnosis of ameloblastoma. An incisional biopsy was obtained confirming the diagnosis of conventional ameloblastoma. Left hemimandibulectomy and plate reconstruction were performed. Histopathological analysis of the surgical specimen confirmed the conventional ameloblastoma with a plexiform pattern and significant areas of cystic degeneration and amyloid-like-like deposits. Additionally, a microarray was carried out with bioinformatic analysis for the enrichment, protein interaction, and determination of eight hub genes (CRP, BCHE, APP, AKT1, AGT, ACTC1, ADAM10, and APOA2) related to their pathogenesis.

## 1. Introduction

The World Health Organization defines ameloblastoma as a benign intraosseous epithelial odontogenic neoplasm, characterized by progressive growth with the destruction of surrounding tissues and a tendency for local recurrence if it is not adequately removed. It was first reported by Cusack in 1827 and named by Ivy and Churchill in 1930 [1,2,3].

An annual incidence of 0.5 cases per million inhabitants is estimated; this makes it one of the most common odontogenic tumors, representing approximately 11% of all odontogenic tumors, with a higher incidence between the fourth and fifth decade of life, without predilection for any gender [4].

Approximately 80% of all ameloblastomas are located in the posterior region of the mandible, followed by the anterior mandibular region, and posterior and anterior maxillary segments. Involvement of the sinus and nasal cavity regions is rare but has been reported [1,5,6,7].

Clinically, it presents as an asymptomatic growth or volume increase accompanied by expansive bone destruction or even cortical perforation. It is common for its early detection to be conducted by imaging finding, observing multilocular or unilocular radiolucent lesions. When the tumor reaches large proportions, deformity, masticatory dysfunction, facial asymmetry, opening limitation, or airway obstruction are observed. These features mean the clinical diagnosis will be more predictable; however, at these stages, the prognosis and quality of life of the patient are reserved, since radical surgical intervention is the alternative to his treatment [8,9,10,11,12].

Its etiology and pathogenesis are still a subject of research. It has been considered that the neoplastic transformation of dental lamina remains; enamel organ elements and epithelial lining of some odontogenic cysts or basal epithelial cells of the oral mucosa may be the histological component for their pathogenesis [3,4,13]. Deregulation of the SHH, WNT/β-catenin, and MAPK signaling pathways has been reported to participate in the pathogenesis of ameloblastoma, and even the detection of the BRAF V600E mutation has been associated with more aggressive clinical courses. The identification of molecular profiles has shown the high complexity of ameloblastoma, as occurs with all cancers, which is why the use of high-throughput assays will help to better understand its clinical course [12,14,15].

The bioinformatics methods on microarray profile datasets represents an important opportunity and strategy to correlate the clinical and molecular features with the possible application of therapeutic and/or prognostic biomarkers of ameloblastoma. Through these assays, the existence of differential changes in inflammatory and immunological mechanisms, MAP kinases, and cell cycle deregulations in ameloblastoma has been determined [16,17,18,19]. As we mentioned above, with this large amount of information, it is possible to postulate candidates for new therapeutic alternatives, as well as to establish correlations with clinical and histological variables of this odontogenic tumor. This study aimed to report the clinical, histopathological, and genic microarray findings of conventional ameloblastoma.

## 2. Case Report

**Figure 1:**
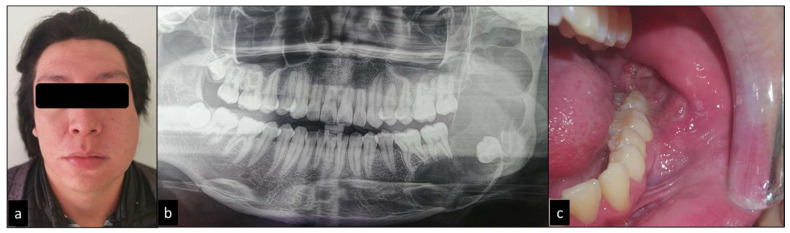
Clinical image. (**a**) Extraoral image shows large and diffuse swelling at middle and lower left facial third causing facial asymmetry. (**b**) Panoramic radiograph image showing a multilocular radiolucent area located in the left mandibular body that extends from the mesial root of the lower left first molar to the ramus of the mandible, with retention of the third molar. (**c**) Intraorally showed expansion of the lingual and vestibular cortices, as well as a polypoid projection in the retromolar area.

A 20-year-old male patient refers to facial asymmetry after 3 months of evolution with a volume increase in the middle and lower left facial third, with painful symptoms. Radiographically, a multilocular radiolucent area was observed in the body, and the entire ascending mandibular branch was associated with the retained third molar. Intraorally, expansion of the lingual and vestibular cortices was observed, as well as a polypoid projection in the left mandibular retromolar area (Figure 1).

The presumptive diagnosis was a probable neoplasm or odontogenic cyst, mainly ameloblastoma versus an odontogenic keratocyst, respectively. An incisional biopsy was performed under local anesthesia, obtaining a specimen of 1.9 × 1.3 × 0.8 cm with irregular edges, a light brown color, and soft consistency. Histopathological analysis revealed an odontogenic neoplasm compatible with conventional ameloblastoma, that presented anastomosing cords organized in a plexiform pattern with lax centers with palisade basal cells immersed over a dense connective tissue stroma.

**Figure 2:**
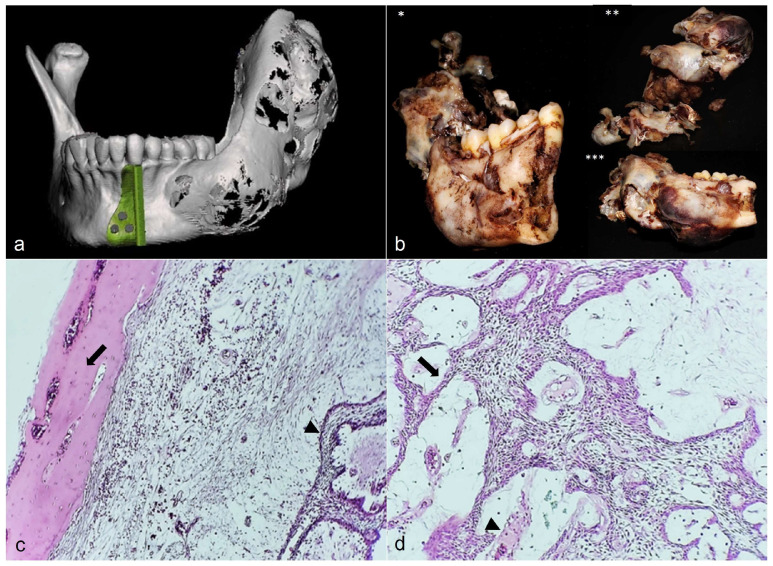
Tomographic surgical planning, specimen, and histopathological images. (**a**) Tomographic 3D reconstruction; (**b**) surgical specimen of approximately 8.0 × 7.5 × 6.6 cm * Vestibular side, ** & *** posterior side with extension to lingual region; (**c**) H&E photomicrography of anterior edge, with presence of bone cortical (arrow), lax stroma, and odontogenic epithelia island and columnar cells of inverted nuclear polarity with central zone that resemble stellate reticulum (arrow head); (**d**) plexiform pattern distribution with anastomosing cords (arrow) with cystic degeneration in a lax stroma, with presence of acellular amyloid-like amorphous eosinophil material (arrow head).

Due to the above histopathological results and the clinical features, it was decided to perform surgical treatment for complete removal of the lesion with safety margins and a definitive histopathological study, for which the patient was sent to the Department of Maxillofacial Surgery of the Balbuena General Hospital to perform the surgical protocol. Through the tomographic study, the extension of the lesion was evaluated; the tumor extension was from the mandibular body at the level of the premolars to the ipsilateral condyle, and it was decided to perform a hemimandibulectomy. Customized cutting guides were made with safety margins of 1.5 cm. For its reconstruction, it was carried out using a designed PMMA 3D customized glenoid fossa with a titanium mandible condyle and a locked 14-hole reconstruction plate 2.4 system. With the previous tomographic study, stereolithography was built to fit the reconstruction plate and evaluate the position of the temporomandibular joint (TMJ) prosthesis. Under balanced general anesthesia, Risdom-type submandibular access was performed to expose the lesion, cutting guides were placed and a reciprocating saw cut was made considering the safety margin, the mandibular segment was extracted with the complete lesion, and a joint prosthesis with the plate was placed. The reconstruction plate was fixed with 3 14 mm locking screws. The surgical sample was sent to the Oral and Maxillofacial Pathology department of the National Autonomous University of Mexico for final study. The final histopathology report of the surgical specimen confirmed the diagnosis of conventional ameloblastoma with a plexiform pattern, presenting significant areas of cystic degeneration, and the presence of an acellular amyloid-like amorphous eosinophil material deposit, with capsule, preserved 0.8 cm from the anterior border and 1.5 cm from the posterior border (Figure 2).

**Figure 3:**
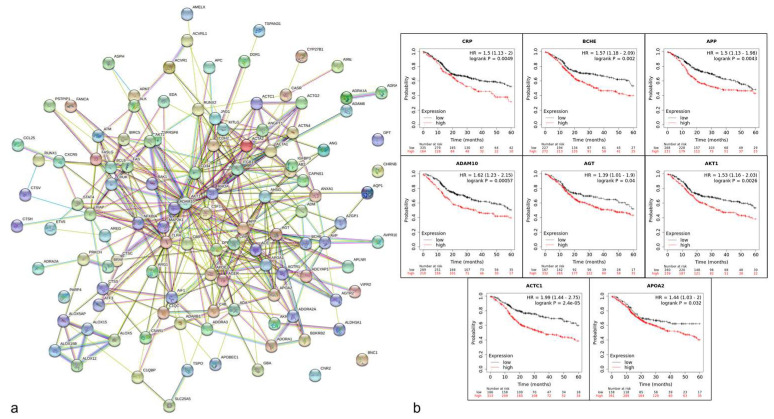
Bioinformatic analysis. (**a**) Network construction of protein-protein interactions. Number of nodes: 116; number of edges: 414; average node degree: 7.14; avg. local clustering coefficient: 0.507; expected number of edges: 157; and PPI enrichment *p*-Value: 1.0 × 10^-16^, (**b**) Kaplan–Meier plots of survival relationship with hub genes obtained, these genes showed an HR >1 and *p* < 0.05.

During the surgical procedure, a representative fragment of the surgical specimen was obtained (1 × 1 × 2 cm approximately) for total RNA extraction by trizol reagent protocol (Invitrogen, Carlsbad, CA, USA) and microarray analysis. An unerupted third molar dental follicle from an 18-year-old patient extracted by orthodontic indication was used as a control. An amount of 2 μg of total RNA from the ameloblastoma and dental follicle were used for the synthesis of cDNA and to perform microarray on the H35K_06_17 AFFYMETRIX Chip, (Santa Clara, CA, USA), the control cDNA was labeled with Alexa 555 and the cDNA ameloblastoma with Alexa 647, followed by mixing and hybridization with the Chip. The overregulated genes were selected through the GenArise software considering only genes with Z > 2 (Supplementary File). Posteriorly R analysis was performed using the Benjamini–Hochberg analysis with a false discovery rate (FDR) *p* < 0.05 as significant. A total of 540 genes were analyzed for the enrichment process by software DAVID 6.8, available online: https://david.ncifcrf.gov (accessed on 7 November 2022), obtaining the differentially expressed genes (DEG) through Gene ontology for biological process (BP), molecular function (MF), and cellular component (CC), and Kyoto Encyclopedia of Genes and Genome selecting only elements statistically significant with a *p* < 0.05 [20]. Subsequently, 116 DEG were obtained to determine the protein–protein interaction, employing the STRING database; version 11.0, http://string-db.org/ (accessed on 7 November 2022) [21]. The interaction network was analyzed using the Cytoscape software with the MCODE application, selecting only genes with a score ≥ 2.5, and selecting 39 genes. To determine their clinical relationship with survival, Kaplan Meier online analysis was conducted (KM plotter, http://kmplot.com/analysis (accessed on 7 November 2022), adjusting the follow-up threshold to 60 months with head and neck squamous carcinoma of overall survival considering an HR > 1 and a *p* < 0.05 [22]. The main genes obtained from the bioinformatic analysis were ACTC1, ADAM10, AGT, AKT1, APOA2, APP, BCHE, and CRP (Table 1). These 8 genes were analyzed through the Metascape system [23], to determine their association with human genetic diseases (DigGenNet), observing links with Senile Plaques and Amyloidosis (Figure 3).

**Figure 4:**
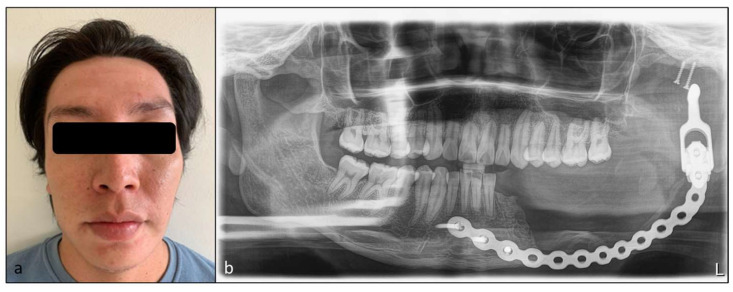
Clinical and imaging status at 15 months of following. (**a**) Extraoral photography of patient, showing symmetric facial appearance without the presence of tumoral remanent. (**b**) Panoramic radiography confirming the absence of tumoral remanent and the correct stability of reconstruction bar and personalized PMMA glenoid cavity.

Currently, with 15 months of evolution, the patient shows adequate facial contour and symmetry. The control orthopantomography showed complete resection of the lesion, without data of its permanence or recurrence, and reconstruction material in adequate position and function, waiting for prosthetic rehabilitation (Figure 4).

## 3. Discussion

According to the World Health Organization in 2017, ameloblastomas are classified into (1) conventional ameloblastoma (previously known as “solid/multicystic ameloblastoma”) which includes patterns: follicular, plexiform, acanthomatous, acanthomatous histopathologic variants, granular cell, basal cell, and desmoplastic; (2) unicystic ameloblastoma with luminal, intraluminal, and mural variants; (3) extraosseous/peripheral ameloblastoma; and (4) malignant or metastatic ameloblastoma. Histologically, it is characterized by tumor nests that resemble the epithelial component of the enamel organ, columnar to cuboidal cells with hyperchromatic nuclei arranged in a palisading pattern with reverse polarity, and the central core is reminiscent of stellate reticulum [1,4,24,25,26].

The clinical course of odontogenic tumors in most cases is indolent, that is, the patient becomes aware of their presence when he observes increases in volume that affect swallowing or phonation, or due to a radiological finding that presents an important affection of maxilla or mandible. When this occurs, the treatment alternative is surgical [27]. Surgical treatment of ameloblastoma could be divided into conservative and radical approaches. In the conservative approach, enucleation is included, and in the radical approach, excision and local reconstruction are considered. Its recurrence is a common complication of conservative treatment; although the conservative approach maintains the integrity of the bone with its growth pattern, it is associated with a high possibility of recurrence ranging from 55–90% [19,28,29,30,31]. To reduce these recurrences, complete bone resection with a safety margin has been considered an adequate option, mainly through marginal or segmental osteotomy for the mandible and partial or total maxillectomy for the maxilla. However, this therapy entails facial sequelae, such as aesthetic and functional deformity, as well as psychological problems in the patient due to radical interventions. Quality of life decreases due to these characteristics [32]. There are various protocols reported for the reconstruction of hemimandibulectomies with condylar extension, the first reported was the costochondral rib graft; however, the metal bars or plates that replace the mandibular body have increased their use frequency [33,34]. Thanks to imaging advances, the computer-assisted and template-guided mandibular reconstruction provided higher accuracy and less variation [35]. In our case, the stereolithography obtained from the tomographic study allowed performing of the plate and evaluation of the position of the TMJ prosthesis, which helps to improve surgical times. The TMJ replacement systems are the complement for the integral reconstruction, and within the reported materials are stainless steel, titanium, and UHMWPE [36]. To our knowledge, this is the first time a designed PMMA 3D customized glenoid fossa has been reported.

Identification of clinical-surgical factors for recurrence, as well as the histological, cellular, and/or molecular understanding, could help in the construction of better clinical, prognostic, and follow-up decisions [37,38,39,40]. The histopathological analysis trying to associate the follicular, plexiform, acanthomatous, or other patterns with their pathogenesis or prognosis is controversial. Thanks to technological advances, principally by high-throughput assays such as genic microarray and bioinformatic analysis, our knowledge about ameloblastoma could improve. Our bioinformatic results confirm that many genes are involved in the pathogenesis of ameloblastoma, such as RUNX1, which had been reported as an indicator of an early epithelial lineage [1]. With our complete bioinformatic analysis, we obtain eight genes that could be related to the tumor development and possible senile plaques and amyloidosis. It was reported that the presence of amyloid deposits can occur in different types of odontogenic cysts and tumors, mainly in epithelial calcifying odontogenic tumors where the amyloid-like presence has been indirectly associated with ameloblastic differentiation and a lack of mineralizing potential. Ameloblastoma is consistent considering ameloblastic differentiation [41,42]. By immunohistochemical reports, the amyloid-like material has been detected by the presence of amelogenins and/or cytokeratins proteins within its composition [43,44]. However, this amyloid-like non-mineralizing protein deposit is an interesting feature, since from the histological and pathogenic point of view it is necessary to investigate whether it has any relationship with cystic degeneration, cell proliferation, or differentiation. Our Kaplan–Meier analysis suggested that high expression of these genes might be associated with poor survival. With these bioinformatic analyses it is possible that pathogenic ameloblastoma development involve these genes in intracellular regulation (AKT1, ACTC1, ADAM10, and APOA2) or for tumor microenvironment regulation (CRP, BCHE, APP, AGT). However, only AKT1 has reports to indicate their association with the regulation of TNFalpha-AKT-MAPK pathway signaling, which promotes cell survival and proliferation in ameloblastoma. It has even been considered that its inhibition could be a therapeutic strategy to promote apoptosis and thus reduce the tumor growth potential, which would have an impact on the design of less aggressive surgical strategies [45,46]. Therefore, to confirm whether these markers are important in the pathogenesis or survival of patients with ameloblastoma, it is necessary to carry out studies with a larger number of patients and with a representative follow-up.

Post-surgical follow-up of patients with hemimandibulectomies must determine the postoperative facial profile and contour in a clinical and radiographical approach along with the facial contour symmetry and occlusion analysis [47]. By 15 months our patient has presented a good state; however, it is necessary to continue with his clinical and radiographic follow-up to be able to intervene in any complication, either due to tumor recurrence or failure in the reconstruction prosthesis.

## 4. Conclusions

The recurrence potential observed in ameloblastoma always makes one think about the best surgical alternative to use. Thanks to the selection of hemimandiulectomy at 15 months of follow-up, no recurrence is observed. The reconstruction through a bar and PMMA personalized glenoid cavity have shown an acceptable state. However, longer-term follow-up is necessary, to minimize side effects and aesthetic and functional complications.

In this case report, it is the first time that a designed PMMA 3D customized glenoid fossa as part of the TMJ replacement systems has been reported, as well as the use of bioinformatic analysis of the gene microarray as a mechanism to understand the clinical and histopathology findings presented in this case. The histopathological analysis is essential not only for diagnosis and therapeutic planning but also as a prognostic factor. The detailed histological analysis of odontogenic tumors is mandatory, considering neoplastic parenchyma and stroma, as well as its relationship with adjacent structures through the surgical edges, which confirms that this analysis is a necessary tool. Using new analysis techniques such as gene microarrays, the information of thousands of genes can be obtained; it is the task of bioinformatics to put an order in these results. Thanks to new databases, bioinformatic estimation for the association of these results with new variables opens the door to innovative research. An interesting finding was the histological presence of amyloid-like deposits, which could correlate with the upregulation of the APP gene. In our opinion, the high-throughput analysis as microarray validates the histopathological findings, increasing our internal validity; however, if we want extrapolation, it is necessary for another research design in clinical investigations, maybe a cases and controls study.

## Figures and Tables

**Table 1:** Bioinformatic Selection of Genes.

DEG	Clustered Genes	Poor Survival-Related Genes
ACE, ACTA1, ACTA2, ACTC1, ACTG2, ACTN4, ACVR1, ACVRL1, ADA, ADAM10, ADAM8, ADARB1, ADCYAP1, ADM, ADORA1, ADORA2A, ADORA3, ADRA1A, ADRA1D, ADRA2A, AGER, AGT, AGTR1, AGTR2, AHSG, AIF1, AIRE, AKR1B1, AKT1, AKT2, ALDH3A1, ALK, ALOX12, ALOX15, ALOX15B, ALOX5, ALOX5AP, AMELX, ANG, ANGPT1, ANXA1, APC, APLNR, APOA1, APOA2, APOBEC1, APOE, APP, AQP1, AREG, ARG1, ARNT, ASPH, ATF3, ATM, AVP, AVPR1B, AZGP1, BAK1, BCHE, BCL6, BDKRB2, BIRC5, BNC1, BRAF, C1QBP, C1QC, C4B, C5AR1, C8B, CAPNS1, CASR, CCL25, CD34, CDH2, CHRNB2, CHUK, CNR2, CRP, CSF1, CTSC, CTSH, CTSS, CTSV, CXCR5, CYP27B1, DAB2, DDR1, DPP4, DPT, EDA, ETV5, FANCA, FAS, FASLG, GBA, IGFBP3, ITGB1, JAG1, KITLG, MAP2K1, NFKBIA, PARP4, PRKCH, PSTPIP1, RHOA, RUNX1, RUNX2, SLC25A5, STAT4, TLR4, TNFRSF8, TSPAN31, TSPO, VIPR2, XIAP	ACE, APOA1, APOE, CRP, BCHE, APP, AGER, ACTA2, TLR4, AKT1, AGTR1, RHOA, AGT, ACTN4, CDH2, ACTC1, ACTG2, JAG1, ADA, ADORA1, ADORA3, ADAM10, C1QC, APOA2, AHSG, C4B, CTSS, ATM, FAS, FASLG, CHUK, XIAP, ALOX5, BCL6, ALOX15, ALOX12, ALOX5AP, ALOX15B, C5AR1	ACTC1, ADAM10, AGT, AKT1, APOA2, APP, BCHE, CRP

Differentially expressed genes (DEG) were obtained from enrichment with Gene ontology and Kyoto Encyclopedy of Genes and Genome; clustered genes were derived from Cytoscape and MCODE analysis; and the poor survival-related genes were selected through Kaplan Meier online analysis. The reduction in the number of genes aims to show which are the central genes for the pathogenesis in this case of ameloblastoma through its bioinformatic analysis.

## Data Availability

The data that support the findings of this study are openly available in the Microarray Unit, Cellular Physiology Institute at http://microarrays.ifc.unam.mx/downloads/Luis_Jacinto_Aleman_FO_UNAM/H35K_06_17 (accessed on 29 July 2022), reference number H35K06 17. The data are not publicly available due to privacy or ethical restrictions.

## Supplementary Materials

The following supporting information can be downloaded at: https://www.mdpi.com/article/10.3390/diagnostics12123190/s1. H35K_06_17_greater_2_Up.
